# Brain Parenchymal Fraction in Healthy Adults—A Systematic Review of the Literature

**DOI:** 10.1371/journal.pone.0170018

**Published:** 2017-01-17

**Authors:** Mattias Vågberg, Gabriel Granåsen, Anders Svenningsson

**Affiliations:** 1 Department of Pharmacology and Clinical Neuroscience, Umeå University, Umeå, Sweden; 2 Epidemiology and Global Health Unit, Department of Public Health and Clinical Medicine, Umeå University, Umeå, Sweden; 3 Department of Clinical Sciences, Karolinska Institutet, Danderyd Hospital, Stockholm, Sweden; Linköping University, SWEDEN

## Abstract

Brain atrophy is an important feature of many neurodegenerative disorders. It can be described in terms of change in the brain parenchymal fraction (BPF). In order to interpret the BPF in disease, knowledge on the BPF in healthy individuals is required. The aim of this study was to establish a normal range of values for the BPF of healthy individuals via a systematic review of the literature. The databases PubMed and Scopus were searched and 95 articles, including a total of 9269 individuals, were identified including the required data. We present values of BPF from healthy individuals stratified by age and post-processing method. The mean BPF correlated with mean age and there were significant differences in age-adjusted mean BPF between methods. This study contributes to increased knowledge about BPF in healthy individuals, which may assist in the interpretation of BPF in the setting of disease. We highlight the differences between post-processing methods and the need for a consensus gold standard.

## Introduction

Pathological loss of brain parenchyma due to neurodegeneration, i.e. brain atrophy, is an important aspect of many diseases affecting the central nervous system (CNS), such as multiple sclerosis,[1] dementia [2] and Huntington’s disease [3]. It is of interest both in the clinical setting [4, 5] and in research and is frequently measured as an endpoint in studies of neurodegenerative diseases.

In order to lessen the impact of inter-individual variation in the amount of brain parenchyma, the volume of parenchyma can be normalized to the size of the intracranial cavity. The intracranial volume (ICV) remains stable during the adult life [6, 7] and can thus be used as a normalization factor independent of age. This normalization can be performed by calculating the ratio of brain parenchymal volume (BPV) to ICV. This ratio was, to the best of our knowledge, initially termed percentage of brain parenchyma [8]. The ratio is now often referred to as brain parenchymal fraction (BPF) after work done by Rudick et al. [1]. Although the definition presented above is the most widely used, small variations in the definition of the term can be seen.

In order to be able to properly interpret levels of BPF in the setting of disease, thorough knowledge of the values in a non-diseased state is necessary.

The goal of this study was to present cross-sectional data on BPF in neurologically healthy individuals via a systematic review of the literature and to relate these values to the ages of the participants and to the method used for the brain segmentations. The primary endpoint for the study was a meta-regression of mean BPF in relation to age. Secondary endpoints were differences in age-adjusted mean BPF between different post-processing methods and method-specific regressions of mean BPF in relation to age.

## Materials and Methods

### Ethics

Since the study only encompasses a review of the literature, it was not subjected to review by an ethical review board.

### Systematic review

The literature search was performed in the two databases PubMed and Scopus on the 7^th^ of January 2016, with the following search string:

("brain parenchymal fraction" OR BPF) AND (“MRI” OR "magnetic resonance imaging" OR “MRT” OR "magnetic resonance tomography" OR “MR” OR “CT” OR "computed tomography")

To increase sensitivity for studies presenting the required data but having used a different terminology than BPF, we redid the search adding a second, separate search string. Keeping the second part of the search string identical (“MRI” OR “magnetic resonance…” etc.) we replaced the first part with “brain volume”. Due to the high number of search results (>20,000) we narrowed it down to (“brain volume” AND (“fraction” or “fractional”)). This still generated a high number of search results (>2,000). Therefore, we further narrowed the search done with this string by restricting it from “all fields” to “article title, abstract, keywords”.

The search was then redone on the 5^th^ of June 2016 with the two search strings:

1. All fields: ("brain parenchymal fraction" OR BPF) AND (“MRI” OR "magnetic resonance imaging" OR “MRT” OR "magnetic resonance tomography" OR “MR” OR “CT” OR "computed tomography")
2. Article title, abstract, keywords: ("brain volume" AND (“fraction” OR “fractional”)) AND (“MRI” OR "magnetic resonance imaging" OR “MRT” OR "magnetic resonance tomography" OR “MR” OR “CT” OR "computed tomography")

**Fig 1:**
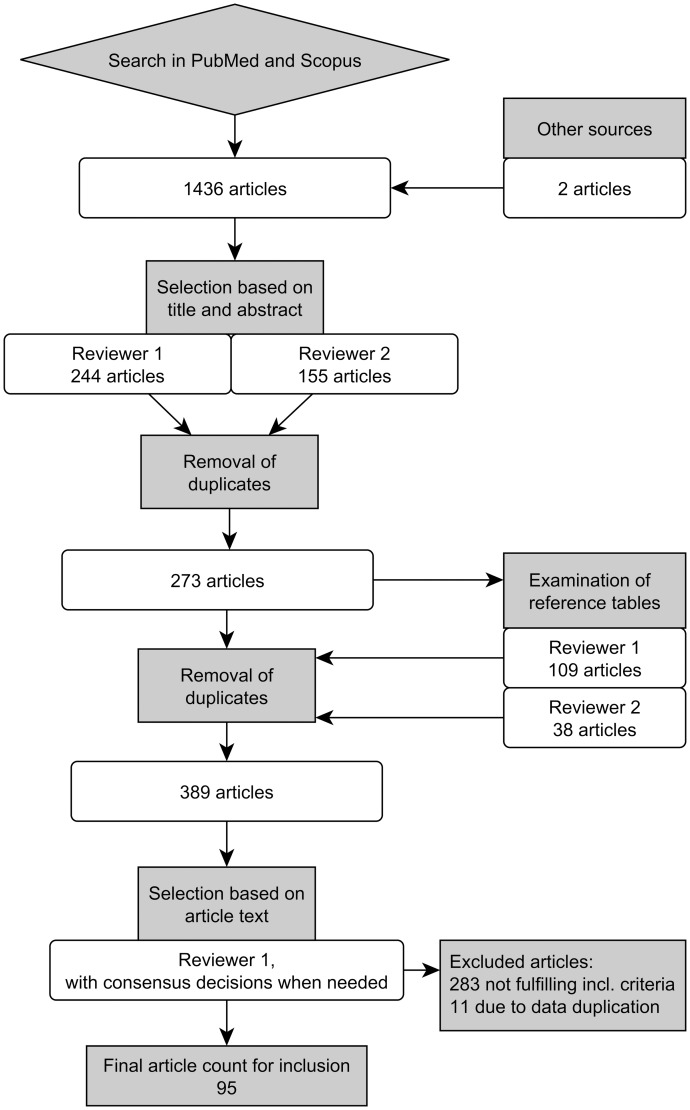
The literature review process visualized by a flow chart.

The literature search is outlined in Fig 1. The numbers described here and in Fig 1 represent the combined results of the two search dates, with duplicates removed. A total of 1434 articles were found in the initial search and two additional articles were added due to personal knowledge, resulting in 1436 articles. Two reviewers (M.V. and A.S.) examined the titles and abstracts of these and 273 articles were found to likely present required data. The reference tables of these 273 articles were examined by both reviewers, and all articles with titles indicating measurement of brain atrophy or brain size in healthy individuals were selected. This yielded 116 new articles, resulting in a total of 389 articles for final examination. The full article texts of these were examined by one reviewer (M.V.) for inclusion according to the criteria below.

The study should be written in English and present:

- data for an adult population (age > 18 years old) free from neurological disease
- age of the study population
- data on BPF or data that enables the calculation of BPF
- BPF measured across the whole intracranial cavity using either MRI or CT

Any uncertainties regarding inclusion were brought up to consensus discussion between the two reviewers. In cases where it was confirmed or highly likely that two or more articles presented data from the same subjects only one of the studies was included. Data on BPF, age, imaging modality and method to determine BPF were extracted from each article.

In cases when the study population was presented as two or more individual subgroups and BPF and age were available for each, the data from each subgroup was extracted instead of the combined data of the whole study population. This allowed for a higher number of age-specific data points and thus a better stratification of BPF by age.

In cases where further specification regarding the methodology would have been of value, an attempt was made to contact the corresponding author to clarify [9–15]. We were able to get in touch with two of the corresponding authors [9, 10] and assessed the methodology of the rest of the mentioned articles according to our best effort.

In cases where an article had determined the BPF in the same population two or more times using different segmentation methods, only one set of BPF data was entered into this review [2, 16, 17]. If the same method had been applied using different MRI scanners, values presented here represents the mean from those measurements [18].

### Definition of BPF

The term BPF was originally presented as the ratio between the BPV and the total volume contained within the brain surface contour (BSC) [1]. The term has later been used both according to the original definition and also to represent the ratio between the BPV and the ICV, the latter now having become predominant.

### Statistics

The statistical calculations were performed in SPSS23 (2015, IBM Corp. Armonk, NY, US), Microsoft Excel 2013 (2012, Microsoft Corporation, Redmond, WA, US) and R 3.2.5 (2016, R Core Team, Vienna, Austria). Visual inspection of histograms in conjunction with Shapiro-Wilks test was used to assess normality of distribution.

In cases when an article included for review did not present the value for BPF, but presented data from which BPF could be calculated, the BPF was calculated together with an estimation of the standard error (SE) in accordance with standard calculation of error propagation [19]. It is noted in Table 1 for which of the studies such calculations were made.

**Table 1:** All the studies included in the review. The individual studies included in the review, subgrouped when applicable. The age and BPF are mean values of the study population.

First author	Subgroup	N(Female)	Age (SD)	BPF (1.96 SE)	Method	BPF-definition
Abe[51]	-	42(20)	48.0(13.2)	0.832(0.034)	SPM	BPV/ICV
Bagnato[7]	-	12(6)	41.5(11.2)	0.850(0.0057)	Other	BPV/ICV
Baltruschat[10]	-	15(7)	30.5(5.9)	0.860(0.0081)	SPM	BPV/ICV
Benedict[52]	-	16(11)	38.3(9.6)	0.884(0.017)	Other	BPV/ICV
Bermel[53]	-	17(13)	35.6(9.9)	0.877(0.0095)	Other	BPV/ICV
Blatter*[54]	1	20(20)	21.3(2.5)	0.936(0.037)	Other	BPV/ICV
	2	24(24)	30.7(3.1)	0.928(0.044)	Other	BPV/ICV
	3	22(22)	40.7(3.0)	0.918(0.045)	Other	BPV/ICV
	4	24(24)	50.4(2.7)	0.913(0.057)	Other	BPV/ICV
	5	15(15)	59.8(2.2)	0.890(0.046)	Other	BPV/ICV
	6	24(0)	23.2(1.9)	0.946(0.036)	Other	BPV/ICV
	7	19(0)	30.9(3.3)	0.927(0.032)	Other	BPV/ICV
	8	16(0)	41.1(2.7)	0.910(0.043)	Other	BPV/ICV
	9	15(0)	51.1(2.7)	0.911(0.044)	Other	BPV/ICV
	10	15(0)	60.6(3.0)	0.877(0.045)	Other	BPV/ICV
Calabrese[15]	-	40(25)	36.2(10.2)	0.854(0.0059)	Other	BPV/ICV
Carone[55]	-	39(26)	39.4(11.5)	0.826(0.0072)	SPM	BPV/ICV
Chard‡[20]	-	29(16)	36.7(N/S)	††0.850(0.78–0.87)	SPM	BPV/ICV
Chen[56]	1	1(1)	33.0(0)	0.871(0.0055)	Other	BPV/ICV
	2	1(0)	33.0(0)	0.894(0.0014)	Other	BPV/ICV
Ciarmiello*[57]	-	54(16)	36.8(13.9)	0.900(0.0064)	Other	BPV/ICV
Cohen[58]	-	19(8)	35.2(10.5)	0.892(0.014)	Other	BPV/ICV
Cruz-Gomez[59]	-	18(18)	31.1(5.7)	0.860(0.0069)	SPM	BPV/ICV
Davies[60]	-	19(10)	34.0(N/S)	0.849(0.0072)	SPM	BPV/ICV
Davies[61]	-	17(10)	35.9(N/S)	0.835(0.013)	SPM	BPV/ICV
De Masi‡[11]	-	12(5)	41.8(10.6)	0.840(0.028)	Other	BPV/BSC
De Andrade[62]	-	10(9)	40.8(3.9)	0.840(0.012)	Other	BPV/ICV
DeCarli‡[32]	1	948(0)	62.2(10.1)	0.772(0.0023)	Other	BPV/ICV
	2	1133(1133)	62.5(10.7)	0.780(0.0019)	Other	BPV/ICV
Delano-Wood*[63]	-	20(12)	78.3(6.3)	0.802(0.040)	Other	BPV/ICV
Dell'Oglio[64]	-	30(21)	43.9(6.3)	0.846(0.0060)	SPM	BPV/ICV
Duning‡[21]	-	65(31)	††61.0(46.0–77.0)	0.787(0.0063)	SIENAX	BPV/ICV
Engström[65]	-	19(14)	48.8(12.2)	0.899(0.011)	SyMap	BPV/ICV
Enzinger[66]	-	201(96)	59.8(5.9)	0.800(0.0028)	SIENAX	BPV/ICV
Fisher‡[31]	-	17(10)	41.6(8.1)	0.862(0.0057)	Other	BPV/BSC
Fisniku*[67]	-	25(14)	41.7(7.7)	0.800(0.0055)	SPM	BPV/ICV
Garcia-Lazaro*[68]	1	21(16)	25.7(3.0)	0.740(0.033)	SPM	BPV/ICV
	2	10(7)	70.2(4.0)	0.678(0.050)	SPM	BPV/ICV
Ge*[69]	1	32(N/S)	33.0(8.9)	0.891(0.020)	Other	BPV/ICV
	2	22(N/S)	67.0(10.4)	0.816(0.022)	Other	BPV/ICV
Ge[70]	-	10(4)	**40.3(23.0–56.0)	0.883(0.021)	Other	BPV/ICV
Glodzik[71]	-	102(38)	72.4(8.3)	0.746(0.0085)	Other	BPV/ICV
Good*[2]	-	10(5)	60.0(6.0)	0.788(0.021)	VBM	BPV/ICV
Good*[72]	1	200(200)	33.5(13.6)	0.740(0.0091)	VBM	BPV/ICV
	2	265(0)	30.9(11.1)	0.764(0.0077)	VBM	BPV/ICV
Gordon-Lipkin [73]	-	15(9)	33.0(7.3)	0.782(0.015)	SIENAX	BPV/ICV
Granberg‡[27]	-	23(18)	57.0(7.2)	0.700(0.0090)	Freesurfer	BPV/ICV
Granberg‡[26]	-	5(2)	51.0(9.9)	†0.785(0.118)	Freesurfer	BPV/ICV
Guttmann*[35]	1	10(4)	23.3(7.6)	0.929(0.019)	Other	BPV/ICV
	2	9(7)	45.6(2.7)	0.901(0.013)	Other	BPV/ICV
	3	8(4)	55.0(2.3)	0.894(0.014)	Other	BPV/ICV
	4	23(16)	66.0(2.9)	0.877(0.012)	Other	BPV/ICV
	5	22(19)	73.5(3)	0.866(0.0088)	Other	BPV/ICV
Harris[74]	-	57(15)	31.5(7.9)	0.935(0.0068)	Other	BPV/ICV
Henkel[75]	-	15(3)	36.0(8.0)	0.837(0.014)	SPM	BPV/ICV
Horsefield[33]	1	1(0)	21.0(0)	0.893(0.027)	Other	BPV/ICV
	2	1(0)	41.0(0)	0.875(0.025)	Other	BPV/ICV
Houtchens[76]	-	16(12)	46.5(9.3)	0.883(0.012)	Other	BPV/ICV
Hsu*[77]	-	893(472)	65.8(9.7)	0.809(0.0077)	VBM	BPV/ICV
Inglese*[78]	-	41(27)	**37.0(23.0–56.0)	0.892(0.018)	Other	BPV/ICV
Janssen[36]	-	40(N/S)	50.7(11.0)	0.827(0.0068)	VBM	BPV/ICV
Jäncke*‡[28]	1	275(275)	24.6(5.7)	0.720(0.0091)	Freesurfer	BPV/ICV
	2	258(0)	26.8(5.6)	0.720(0.011)	Freesurfer	BPV/ICV
	3	177(177)	67.8(8.5)	0.686(0.012)	Freesurfer	BPV/ICV
	4	146(0)	70.8(6.2)	0.669(0.015)	Freesurfer	BPV/ICV
Kalkers‡[22]	-	12(7)	42.5(7.8)	†0.87(0.83–0.88)	Other	BPV/ICV
Kassubek[79]	-	22(5)	59.0(11.0)	0.803(0.015)	SPM	BPV/ICV
Kaussbek[3]	-	70(27)	42.7(15.8)	0.828(0.0086)	SPM	BPV/ICV
Kearney[80]	-	28(19)	41.4(10.3)	0.823(0.0056)	SPM	BPV/ICV
Kilsdonk‡[29]	-	11(6)	38.8(10.5)	0.650(0.024)	Freesurfer	BPV/ICV
Klawiter[81]	-	21(16)	44.0(6.8)	0.846(0.064)	SPM	BPV/ICV
Knutson*[82]	1	38(0)	29.4(6.7)	0.832(0.029)	Other	BPV/ICV
	2	48(48)	31.4(7.5)	0.825(0.030)	Other	BPV/ICV
Kruggel[37]	1	254(0)	27.3(10.2)	0.877(0.0022)	Other	BPV/ICV
	2	248(248)	30.0(9.6)	0.873(0.0021)	Other	BPV/ICV
Leigh[34]	-	5(2)	34.8(10.4)	0.882(0.019)	Other	BPV/ICV
Lemaitre*[83]	1	331(0)	69.5(3.1)	0.734(0.0029)	VBM	BPV/ICV
	2	331(331)	69.6(2.95)	0.747(0.0031)	VBM	BPV/ICV
Liptak[84]	-	29(22)	46.2(12)	0.870(0.015)	Other	BPV/ICV
Liu[85]	-	35(28)	32.2(10.13)	0.860(0.0033)	SPM	BPV/ICV
Lukas[86]	-	3(2)	32.0(4.6)	0.863(0.019)	Other	BPV/ICV
Marquis*[87]	-	60(34)	80.7(8.2)	0.787(0.026)	Other	BPV/ICV
Matsumae[88]	1	12(6)	35.0(6.0)	0.930(0.0057)	Other	BPV/ICV
	2	15(5)	50.0(6.0)	0.910(0.010)	Other	BPV/ICV
	3	22(12)	72.0(5.0)	0.850(0.012)	Other	BPV/ICV
Metzler-Baddeley[89]	-	39(22)	67.6(8.6)	0.692(0.013)	SPM	BPV/ICV
Mezzapesa[90]	-	9(3)	51.8(12.7)	0.849(0.012)	SIENAX	BPV/ICV
Minnerop[91]	-	13(5)	53.5(10.2)	0.660(0.022)	VBM	BPV/ICV
Mitchell[92]	1	290(0)	76.0(4.0)	0.710(0.0046)	Other	BPV/ICV
	2	378(378)	75.0(4.0)	0.750(0.0040)	Other	BPV/ICV
Moriya*[93]	-	19(10)	29.7(11.3)	0.790(0.041)	VBM	BPV/ICV
Metzler-Baddeley[89]	-	39(22)	67.6(8.6)	0.692(0.013)	SPM	BPV/ICV
Moscufo[94]	-	33(14)	82.0(4.4)	0.714(0.013)	Other	BPV/ICV
Oliveira[95]	-	10(9)	40.8(3.9)	0.840(0.012)	Other	BPV/ICV
Pardini[9]	-	22(13)	44.4(2.4)	0.840(0.0084)	Other	BPV/ICV
Peinemann[96]	-	25(12)	42.9(9.8)	0.824(0.018)	VBM	BPV/ICV
Quarantelli*[97]	-	54(32)	38.5(13.1)	0.909(0.0064)	Other	BPV/ICV
Reinhard[98]	-	26(5)	52.0(15)	0.820(0.019)	SIENAX	BPV/ICV
Riello*[99]	1	71(0)	58.9(11.6)	0.807(0.0091)	VBM	BPV/ICV
	2	158(158)	57.2(10.3)	0.828(0.0066)	VBM	BPV/ICV
Rudick‡[1]	-	16(11)	32.3(7.1)	0.871(0.0039)	Other	BPV/BSC
Sanfilipo[100]	-	18(12)	36.2(8.9)	0.882(0.014)	SPM	BPV/ICV
Sastre-Garriga‡[23]	-	45(22)	††39.0(23–67)	0.830(N/S)	SPM	BPV/ICV
Sharma*[16]	-	17(12)	35.9(8.9)	0.880(0.014)	SPM	BPV/ICV
Smith[101]	-	67(40)	71.2(4.4)	0.809(0.0093)	Other	BPV/ICV
Stosic[43]	-	49(33)	44.0(10.9)	0.829(0.0056)	SPM	BPV/ICV
Streitberger‡[12]	-	38(22)	48.0(9.7)	††0.980(0.96–0.99)	SIENAX	BPV/ICV
Tavazzi[102]	-	38(21)	33.3(11.5)	0.848(0.0032)	SIENAX	BPV/ICV
Tiberio[103]	-	10(4)	37.1(N/S)	0.836(0.017)	SPM	BPV/ICV
Tisell‡[24]	-	20(15)	58.8(14.0)	††0.860(0.78–0.94)	SyMap	BPV/ICV
Torelli[94]	-	14(5)	57.6(5.2)	0.853(0.012)	Other	BPV/ICV
Tovar-Moll[13]	-	24(N/S)	41.9(8.3)	0.840(0.0080)	SIENAX	BPV/ICV
Traboulsee[104]	-	63(44)	39.6(9.4)	0.850(0.0049)	SPM	BPV/ICV
Tseng*‡[30]	1	10(3)	72.4(5.6)	0.663(0.040)	Freesurfer	BPV/ICV
	2	10(2)	74.6(4.3)	0.645(0.046)	Freesurfer	BPV/ICV
Uçar[105]	-	10(8)	31.5(5.0)	0.856(0.0064)	Other	BPV/ICV
Vagberg‡[25]	-	35(19)	††35.0(19.0–65.0))	0.890(0.0080)	SyMap	BPV/ICV
Vagberg‡[17]	1	21(9)	†22.8(4.5)	†0.887(0.037)	SyMap	BPV/ICV
	2	20(11)	†33.9(3.1)	†0.870(0.032)	SyMap	BPV/ICV
	3	20(7)	†44.1(4.6)	†0.866(0.029)	SyMap	BPV/ICV
	4	17(12)	†52.6(6.9)	†0.837(0.033)	SyMap	BPV/ICV
	5	15(8)	†67.3(5.8)	†0.811(0.041)	SyMap	BPV/ICV
	6	11(8)	†75.3(4.8)	†0.7780.071)	SyMap	BPV/ICV
	7	2(1)	†82.7(4.5)	†0.697(0.0060)	SyMap	BPV/ICV
Warntjes[42]	-	20(15)	48.0(12.0)	0.885(0.014)	SyMap	BPV/ICV
West[18]	-	10(6)	24.4(2.5)	0.909(0.012)	SyMap	BPV/ICV
Wuerfel[14]	-	30(16)	37.8(11.5)	0.870(0.0072)	SIENAX	BPV/ICV
Yaldizli[106]	-	27(17)	41.7(13.9)	0.771(0.0098)	Other	BPV/ICV
Yamasue*[107]	-	76(38)	41.7(11.9)	0.747(0.023)	VBM	BPV/ICV
Zimmerman[108]	-	20(12)	35.7(9.6)	0.723(0.018)	VBM	BPV/ICV
Zito[109]	-	15(11)	37.2(9.1)	0.820(0.096)	VBM	BPV/ICV
Zivadinov[110]	-	19(10)	30.4(12)	0.845(0.0027)	SIENAX	BPV/ICV

* denotes studies where the BPF was not presented directly but information was available to allow calculation.

** denotes a value that is presented as mean (range)

^†^ denotes a value that is presented as median (interquartile range)

^††^ denotes a value that is presented as median (range)

^‡^ denotes a study that was excluded from the aggregated statistical calculations due to methodology or data presentation

BPF = Brain Parenchymal Fraction

BPV = Brain Parenchymal Volume

BSC = the volume within the Brain Surface Contour

ICV = Intracranial Volume

N/S = Not Specified

SD = Standard Deviation

SE = Standard Error of the Mean

SIENAX = Structural Image Evaluation Using Normalisation of Atrophy Cross-Sectional

SPM = Statistical Parametric Mapping

SyMap = Synthetic Tissue Mapping

VBM = Voxel Based Morphometry

Only studies presenting data on mean BPF and mean age were included in the aggregated statistical calculations. When median age and/or BPF was presented instead of the mean, the median was noted in the study summary in Table 1 but the study was excluded from the aggregated statistical calculations [12, 17, 20–25].

A meta-regression using weighted mixed effects linear regression was used to explore the relationship between mean BPF and age. The age dependency was explained by a linear and a quadratic term. By weighting the observations by the inverse of the standard deviations from the BPF-estimates the analysis handled differences in sample sizes and precision between the studies. A random effect was used for controlling for study specific bias in cases when two or more individual populations originated from the same study.

Spearman’s Rho was used for correlation testing. Estimated marginal means were calculated from the regression models in order to compare age-adjusted mean BPF-levels between methods. Corresponding p-values were adjusted using the Bonferroni-Holm method.

## Results

### Final article inclusion

The final article count for inclusion was 95. The included articles are presented in Table 1. The studies presented data on a total of 131 independent populations amounting to a total of 9269 (4932 female) individuals reported to be neurologically healthy. For the studies reporting mean values, the mean BPF values (n = 119) ranged from 0.645 to 0.946 and the mean ages (n = 121) ranged from 21.0 to 82.0 years. Seventeen [1, 11, 12, 17, 20–32] of the 95 studies are presented in Table 1 but were excluded from part of, or all, of the aggregated statistical calculations on the basis of methodology or data presentation (see the section on statistics and the rest of the results section for further details). The final number of independent populations used for the complete aggregated statistical calculations were 103, presented in 78 studies and amounting to 5878 (3102 female) healthy individuals with mean BPF values ranging from 0.660 to 0.946 and mean ages ranging from 21.0 to 82.0 years.

Two articles had investigated BPF using several different pulse sequences and segmentation methods but only presented the mean values of all of these [33, 34]. One article had determined BPF two consecutive times six days apart and the values presented in this review are the means from those measurements. One article [35] had stated that the data spread was expressed as SE but the values presented were too large to be representing SE in relation to the reported range and were therefore assumed to be standard deviation (SD) instead. One article [12] did not specify if the presented value was median or mean. We decided to include this value in the statistical calculations as if it was the median. In one article the mean age of the population was stated for the whole population (n = 55) and not the subgroup that had undergone MRI (n = 40) [36]. We decided to approximate the mean age of the MRI group with that from the whole population. We decided to include one study that had examined individuals with ages ranging between 16 and 70 years old, but with a mean age and data spread that indicated that the population was predominantly adult [37].

### Imaging modality and method of BPF determination

All articles in the final article count had determined BPF by MRI, using a variety of different post-processing methods. The method used by each study is noted in Table 1, categorized as Structural Image Evaluation Using Normalisation of Atrophy Cross-Sectional (SIENAX) [38], Voxel Based Morphometry (VBM) [39], Synthetic Tissue Mapping (SyMap) [25], Statistical Parametric Mapping (SPM) [40], Freesurfer [41] or “Other”. Freesurfer brain segmentation does not by default include the brainstem. For this reason, the Freesurfer studies [26–30] were excluded from the aggregated statistical analyses. However, since Freesurfer is a well-known segmentation method the studies were presented in Table 1 and included in the method specific calculations of the regression of BPF in relation to age and the estimated marginal means. The SyMap results are specified in Table 1 but were included in the category “Other” for the regression analyses, due to a low number of SyMap data points.

### Definition of BPF

The definition of BPF used in each study included in this review could broadly be categorized as either BPF = BPV/ICV or BPF = BPV/BSC. This categorization is noted in Table 1. Studies using the definition BPF = BPV/BSC (n = 3) were excluded from the statistical analyses. In three cases the exact definition of BPF used was not clearly stated and attempts to contact the corresponding author were unsuccessful [13–15]. In these cases, the definition was assumed to be BPF = BPV/ICV.

There were slight differences among the included studies regarding the details of the calculation of ICV and BPV. As an example, the ICV could be defined as either the intracranial cavity measured directly [42] or as the sum of the volumes of GM, WM and CSF [43]. Only studies having included the complete intracranial space in the assessment of ICV were included in the aggregated statistical calculations. One study that had only reported the supratentorial ICV was entered into Table 1 [32]. Due to the large population size we considered the study important to report, but excluded it from the aggregated statistical calculations.

### BPF in relation to age

**Fig 2:**
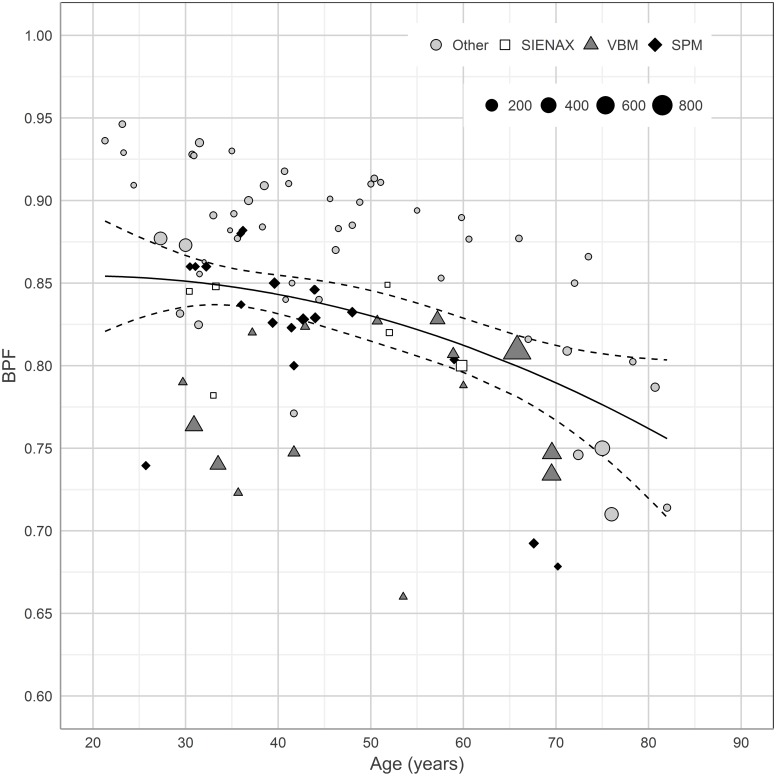
The mean BPF values of the study populations plotted in relation to age. Mean BPF value of each study population (vertical axis) is plotted against mean age (horizontal axis). The regression line is a mixed weighted regression model. The dashed lines represent 95% confidence interval. Seventeen studies were excluded from this figure (see the sections on statistics and results). BPF = Brain Parenchymal Fraction. SIENAX = Structural Image Evaluation Using Normalisation of Atrophy Cross-Sectional. SPM = Statistical Parametric Mapping. VBM = Voxel Based Morphometry.

There was a significant correlation between mean population BPF values and mean population ages (R = -0.41, p<0.001) (Fig 2). When examining the method specific residuals to the regression in Fig 2, all post-processing methods exhibited relatively uniform residuals in relation to mean age with the exception of VBM, where the residuals indicated an age-dependent effect on the fit to the curve.

### BPF determined by different post-processing methods

**Fig 3:**
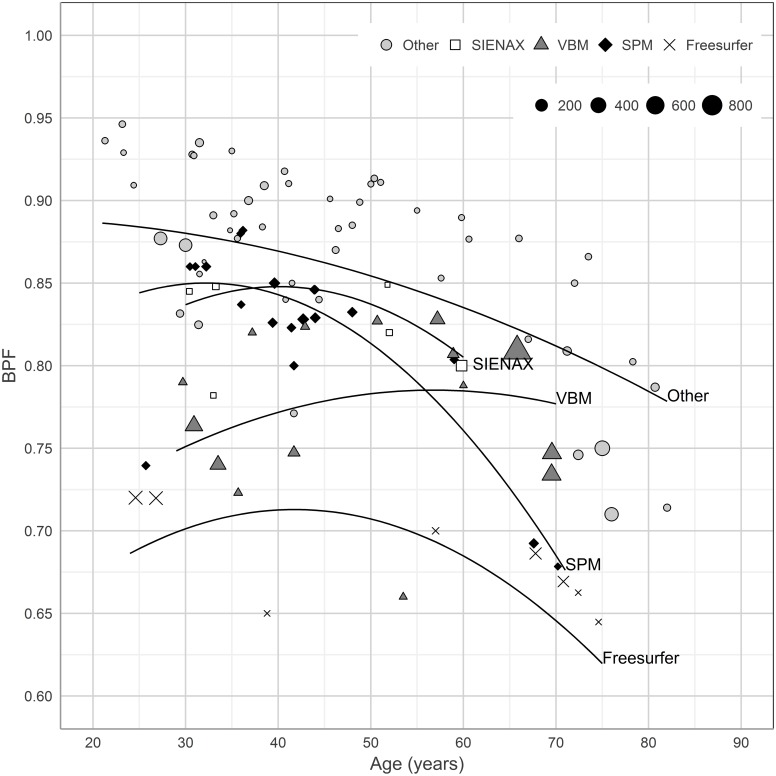
The mean BPF values of the study populations plotted individually for each post-processing method. Mean BPF value of each study population (vertical axis) is plotted against mean age (horizontal axis). Individual mixed weighted regression lines are plotted for each post-processing method. Twelve studies were excluded from this figure (see the sections on statistics and results). BPF = Brain Parenchymal Fraction. SIENAX = Structural Image Evaluation Using Normalisation of Atrophy Cross-Sectional. SPM = Statistical Parametric Mapping. VBM = Voxel Based Morphometry.

Fig 3 shows the BPF of the study populations in relation to population age, stratified by post-processing method. There were significant differences between the different post-processing methods (p≤0.05 for all comparisons) with the exception of the comparison of SIENAX to SPM (p = 0.74). The comparison of “Other” to SIENAX did not retain statistical significance after Bonferroni-Holm adjustment (p = 0.088). The estimated magnitude of difference in age-adjusted BPF between the methods (excluding non-significant differences) ranged between 0.038 and 0.17.

Three studies included in the aggregated statistical calculations had methodological uncertainties, which we did not succeed to clarify by an attempt to contact the corresponding author [13–15]. After exclusion of these studies, the comparison of SIENAX to VBM was still significant (p = 0.044) but did not retain significance after Bonferroni-Holm adjustment (p = 0.11). The three studies were not outliers and no other statistical significances changed if they were excluded.

## Discussion

We here present a database of BPF in healthy individuals, stratified by age and method of segmentation, composed of data from a systematic review of the literature. The data may aid researchers and clinicians in the interpretation of BPF data in relation to method and age.

The regression indicates a progressive rate of atrophy with increasing age. This is supported by earlier findings in individual studies [6, 7, 17, 44, 45]. The biological background for this possibly progressive atrophy rate in healthy individuals is not clear. It could be hypothesized that increased prevalence of subclinical vascular/ischemic tissue damage with increasing age is a factor but more research is needed to elucidate this.

It is also noteworthy, although not surprising, that there seems to be a large degree of heterogeneity between different post-processing methods. Age-adjusted BPF values were significantly different between methods. This accentuates the need for caution when comparing BPF data between studies, especially if different post-processing methods have been used. This also highlights the problem that there is no generally accepted consensus regarding gold standard for BPF determination, or for quantifying brain atrophy at large. A consensus gold standard would simplify validation of new methods and provide a unified reference for discussions regarding brain atrophy.

It is of interest to mention that the BPF of all post-processing methods, with the exception of VBM, could be fitted relatively well to the quadratic mixed weighted regression model from the aggregated data from all populations (Fig 2). However, the VBM residuals indicated an age-dependent bias in the BPF determination if compared to the aggregated data from all methods. The issue of potential age bias in the VBM segmentation, perhaps in part explained by the atlas template used for the segmentation [46, 47], have been discussed previously [17] and is important to note.

A strength of the study is the systematic methodology used in order to maximize the possibility of covering most of the suitable studies in the databases searched. The choice of databases, PubMed and Scopus, encompass a large proportion of published clinical studies and are therefore likely to include most studies presenting BPF in healthy adults. It must be mentioned, however, that there could be studies that would have been suitable for inclusion but were not indexed in these databases.

Another strength of the study is the presentation of method specific regressions of BPF over age. This could benefit researchers in providing literature values to use as a comparison for their own study data. More importantly, it emphasizes the difficulties in comparing data between studies if they are performed with different post processing techniques.

An obstacle to overcome was that other terms than BPF have been used to denote the same ratio. In an effort to identify as many relevant studies as possible, regardless of the terminology used, articles with titles and/or abstracts not specifically mentioning BPF but indicating that brain volume or brain atrophy measurements in healthy individuals had been investigated were also included in this review. Data for calculation of BPF was extracted whenever possible even if BPF was not reported directly in the article. Despite these efforts, however, it is likely that there are studies that have not been found due to usage of different terminology. It is also likely that the review has not identified the entirety of studies presenting suitable data for the calculation of BPF, in place of presenting BPF itself, as we for practical reasons chose to narrow the search from the more sensitive search term “brain volume”.

A limitation of the study is the use of summary statistics from each study population in the form of mean age and BPF. More detailed statistical calculations could have been performed if data on individual values for each study participant had been available.

It is furthermore important to note that we specifically chose to limit this review to the BPF-ratio and that there are studies presenting brain volume or other morphological brain features biologically related to BPF while not presenting the data required for the calculation of BPF. The work by Taki and colleagues is a notable example [48]. We chose to limit this review solely on the BPF to facilitate a focused presentation of data. We also chose to be stringent regarding the measurements on which to base the BPF and chose not to include studies that only reported approximations on or parts of the volumes of interests. An example is the valuable work by Coffey and colleagues reporting brain volumetric data from 330 volunteers but, as the authors point out, having restricted the estimation of the ICV to a part of the true volume [49]. Therefore, we chose not to include it in this review. We made one notable exception to this rule in including the work by DeCarli and colleagues on the Framingham Heart Study, presenting brain volumetric data on ICV and BPV from 2081 individuals [32]. This is an important study to report due to the large population size. However, the measurements of ICV and BPV in the study, by which we calculated the BPF, were restricted to the supratentorial space. We chose to include the data in Table 1 but exclude it from the statistical analyses.

It is necessary to point out that this review only presents cross-sectional data on BPF and large inter-individual variations have been reported for such data [17]. This means that an individual BPF value would need to deviate far from the expected value for the age in order to be certain that the value is abnormal. Longitudinal data on brain atrophy, measured over several time points, provides a different perspective on the development of atrophy. However, the magnitude of physiological effects on brain volume that have been reported, for example in the setting of dehydration-rehydration [50], is not negligible in relation to the expected change in brain volume per year [45], complicating the quantification of annual changes in brain volume. We suggest that a combination of cross-sectional and longitudinal data would provide the most robust assessment of brain atrophy. More research is needed on this subject, especially regarding longitudinal rate of brain volume change in relation to age in the non-diseased state.

## Conclusions

Knowledge of the normal range of brain atrophy assessments in relation to age is important when interpreting brain atrophy data. We believe that this article contributes to a base of such knowledge while we want to point out that further knowledge regarding longitudinal annual atrophy rate in healthy individuals is needed. The data presented here may benefit researchers wanting to compare their own study data to literature values for their chosen method of segmentation. The heterogeneity existing between different methods for BPF determination emphasizes the need for a consensus gold standard.

## Supporting Information

S1 File PRISMA Checklist. (DOC)

## Abbreviations

BPF: Brain Parenchymal Fraction
BPV: Brain Parenchymal Volume
BSC: the volume within the Brain Surface Contour
CT: Computed Tomography
ICV: Intracranial Volume
MRI: Magnetic Resonance Imaging
N/S: Not Specified
SD: Standard Deviation
SE: Standard Error of the Mean
SIENAX: Structural Image Evaluation Using Normalisation of Atrophy Cross-Sectional
SPM: Statistical Parametric Mapping
SyMap: Synthetic Tissue Mapping
VBM: Voxel Based Morphometry
